# Consumption of Coffee and Risk of Gestational Diabetes Mellitus: A Systematic Review and Meta-Analysis of Observational Studies

**DOI:** 10.3389/fnut.2021.739359

**Published:** 2021-09-20

**Authors:** Jiaying Ni, Ping Wang, Tao Zheng, Long Lv, Hao Peng

**Affiliations:** ^1^Department of Obstetrics and Gynecology, Xinhua Hospital Affiliated to Shanghai JiaoTong University School Medicine, Shanghai, China; ^2^Department of Internal Medicine, Nanjing Gaochun People's Hospital, Nanjing, China; ^3^Department of General Surgery, Nanjing Gaochun People's Hospital, Nanjing, China; ^4^Department of Obstetrics and Gynecology, Huangshi Central Hospital, Affiliated Hospital of Hubei Polytechnic University, Edong Healthcare Group, Huangshi, China

**Keywords:** coffee, gestational diabetes mellitus, meta-analysis, systematic review, safety

## Abstract

**Objective:** The results from epidemiologic studies on the relationship between intake of coffee and the risk of gestational diabetes mellitus (GDM) remain inconclusive. A meta-analysis was performed to achieve a comprehensive finding regarding the association between intake of coffee and the risk of GDM.

**Methods:** PubMed, Scopus, ISI Web of Science, and Google Scholar were searched to find articles published up to August 2021. Observational studies that reported risk estimates [risk ratios (RRs), hazard ratios (HRs), and odds ratios (ORs)] for the association of consumption of coffee with the risk of GDM in pregnant women were included. Random effects model was applied to calculate summarized risk estimate and 95% CIs for the highest vs. lowest categories of intake of coffee.

**Results:** Seven observational studies (three cohort, two case-control, and two cross-sectional studies) with 75,607 participants and 1,625 women with GDM met the inclusion criteria. The meta-analysis of comparing the highest vs. lowest intake of coffee categories showed no significant association between intake of coffee and risk of GDM (summarized risk estimate: 0.89; 95% CI: 0.76, 1.05; *I*^2^ = 63.4%). Subgroup analysis showed that consumption of coffee had an inverse relationship with GDM in studies conducted in non-Asia countries (summarized risk estimate: 0.75; 95% CI: 0.58, 0.97; *I*^2^ = 6%).

**Conclusion:** This study has shown that high consumption of coffee did not decrease the risk of GDM. Furthermore, large-scale cohort studies are required to confirm our findings.

## Introduction

Gestational diabetes mellitus (GDM) is a disease in which pregnant women who did not have diabetes before pregnancy develops glucose intolerance because of the interference of pregnancy hormones with the action of insulin (1, 2). It has been estimated that roughly 15 to 20% of pregnant women were affected by GDM (3). High gestational age, obesity, polycystic ovary syndrome, ethnicity, glycosuria, family history of diabetes, and previous history of GDM are underlying risk factors of GDM (4). Approximately 70% of women with GDM will be affected by type 2 diabetes mellitus (T2DM) later in their life (5). Infants born to mothers with GDM are at increased risk of macrosomia, hypoglycemia, jaundice, and epigenetic changes (6). In the long term, they are prone to being obese or diabetic in childhood (7). Therefore, the identification of preventive strategies to reduce GDM has great importance.

Diet and physical activity are fundamental lifestyle interventions to control GDM (7). Using foods with a low glycemic index, high antioxidants, and also decrease in intake of energy, distribution of carbohydrates, and intake of fat or protein modification share some dietary recommendations for women with GDM (8–11).

Coffee is commonly drunk among women aged 20–50 years, and thus their possible effects on GDM absorb many interests (12). A meta-analysis of prospective cohort studies exhibited both the caffeinated and decaffeinated coffee was associated with reduced diabetes risk (13). It appears that the influence of intake of coffee on GDM may vary from T2DM in non-pregnant women. Metabolism of caffeine as the main phytochemical in coffee decreases during pregnancy (14), and subsequently, a high amount of caffeine levels in blood was related to elevating insulin resistance (15). It seems that the favorable effects of coffee are not linked to caffeine and its metabolites during pregnancy, and originated from antioxidants and prebiotic compounds, including phenolic components and micronutrients, which can improve insulin sensitivity and glycemic response (16).

The findings of studies regarding the association of intake of coffee with GDM are conflicting. A double-blind, randomized crossover study indicated that acute caffeine ingestion impairs insulin sensitivity in women with GDM (17). Furthermore, some observational studies displayed no relationship between intake of coffee and GDM (18–21), while two studies showed inverse associations (22, 23).

Based on our knowledge, there is no systematic review and meta-analysis to clarify the association between the consumption of coffee and the risk of GDM. Therefore, the purpose of this study is to achieve a solid response to this question: Is consumption of coffee associated with the risk of GDM in pregnant women?

## Methods

The protocol of this study has been established based on Preferred Reporting Items of Systematic Reviews and Meta-Analysis (PRISMA) criteria (24).

### Search Strategy

Two independent authors undertook a systematic search in PubMed, Scopus, ISI Web of Sciences, and Google Scholar to determine the pertinent articles with publication dates until August 2021. The search was performed using medical subject heading (MeSH) and related keywords including: coffee, caffeine diabetes, gestational, gestational diabetes, GDM or diabetes, and gestational (Mesh). No restrictions such as language were taken into account when the search was conducted. The citations of selected articles and retrieved reviews were manually checked to avoid missing any papers.

### Inclusion and Exclusion Criteria

Studies with the following criteria were eligible for this review: observational studies (cohort, cross-sectional, or case-control studies), those carried out on pregnant women, studies reported risk estimates [relative risk (RR), odds ratio (OR), and hazard ratio (HR) with corresponding 95% CIs] for the association between consumption of coffee and the risk of GDM. Articles were included whether they considered total coffee or caffeinated coffee and decaffeinated coffee separately.

We excluded one study that assessed the association between coffee and tea and the risk of GDM (25). Tea has a different nutritional composition as compared with coffee, and therefore, the compounds in tea are likely to have different effects, and assessment of a combination of coffee and tea unable us to find a pure effect of coffee. Furthermore, irrelevant papers, abstracts, unpublished essays, review articles, commentary, editorial, or letters were removed.

### Quality Assessment

Newcastle-Ottawa Scale (NOS) was used to determine the risk of bias of each article included (26). If one study acquires a score of ≥7, it is contemplated as high quality. Two researchers evaluated the methodological quality of each study independently. If they could not reach any consensus, a third party (Principal investigator) decided by a discussion with them.

### Data Extraction and Abstraction

Two investigators exploited separately the desired information using prespecified forms, and in case when they faced disagreements, they discussed it with the third author to reach a firm opinion. The following data were extracted: surname of the first author, date of publication, study design, geographic region, age, gender, follow-up duration (in prospective cohort studies), sample size, number of cases, number of controls in case-control studies, categories of intake of coffee, estimated risk (RR, HR, OR), diagnostic criteria for GDM, dietary measurement method, and adjusted variables. In the terms of estimated risk with different adjustment models, we choose that one controlled the greatest number of main covariates. If one study reported a separate risk estimate for caffeinated and decaffeinated coffee rather than the total consumption of coffee, we included a risk estimate for caffeinated coffee in the principal analysis.

### Statistical Analysis

In the meta-analysis comparing high vs. low intake categories of coffee, we used a random effects model to combine risk estimates (including RRs, HRs, and ORs) and 95% CIs of GDM. To assess the weight of each study, the standard error for the log RR/HR/OR of each study was regarded as the estimated variance of the log RR, using inverse variance methods (27). Cochrane *Q*-test and *I*^2^-test were used to assess heterogeneity among pertinent studies. Cochrane *Q* test, with *P* < 0.1 indicating significant between-study heterogeneity. The values *I*^2^ of 25–50, 50–75, and >75% were considered as low, moderate, and high heterogeneity, respectively (28). Subgroup analysis was implemented to identify sources of heterogeneity according to the relevant variables: geographic region, study design, number of cases, sample size, diagnostic criteria for GDM, dietary assessment method, adjustment to dietary energy intake, and body mass index (BMI), and quality of studies. Inspection of the funnel plots for asymmetry and Egger test (*P* < 0.10) were employed to detect publication bias (29). A sensitivity analysis was carried out to investigate the dependency of overall effect size to each study by leaving one study and repeating the analysis. All statistical analyses were performed using STATA software version 15.1 (Stata Corporation, College Station, Texas, USA). The *P* > 0.05 was speculated as significant.

## Results

**Figure 1:**
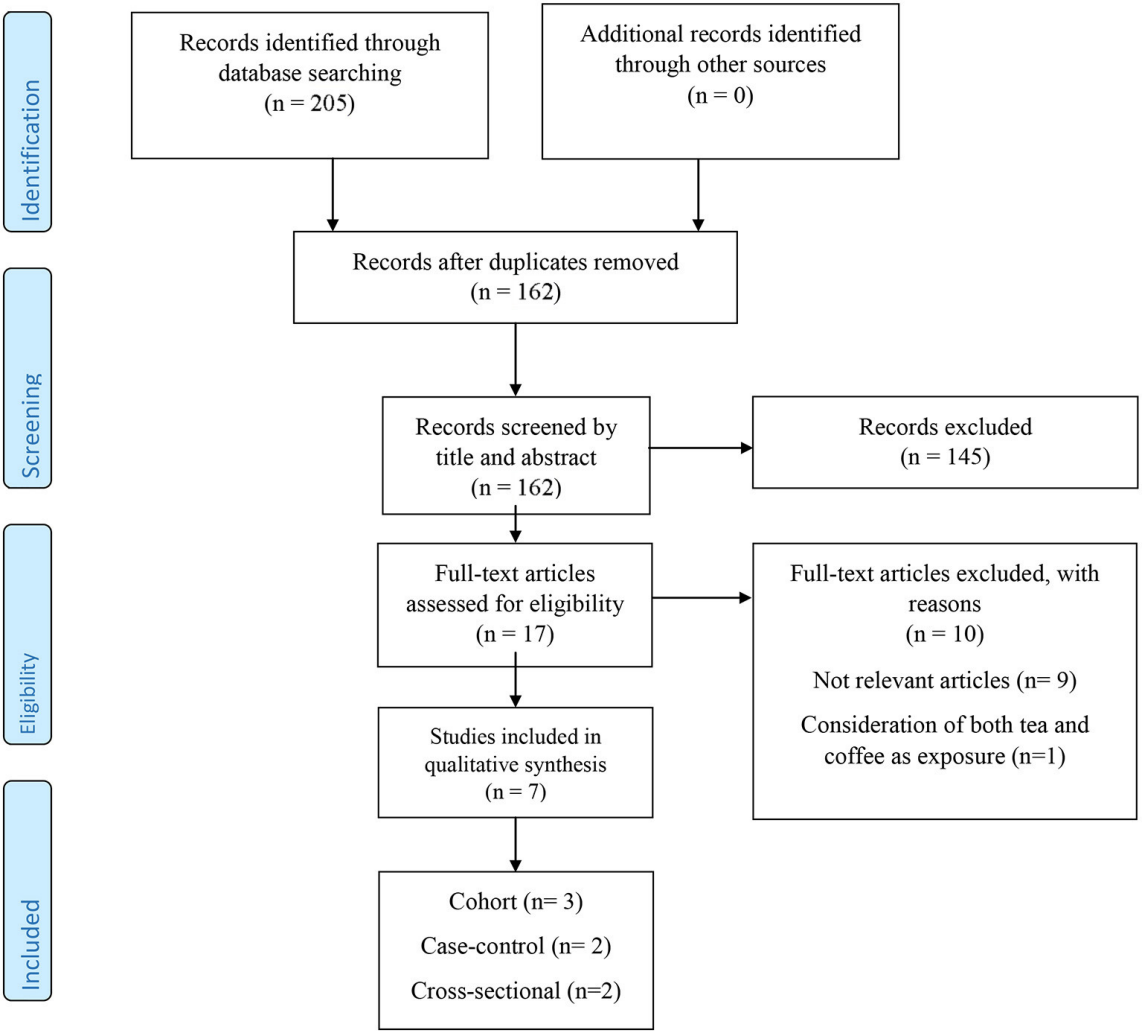
The flow diagram of study selection.

The flowchart of study selection was displayed in Figure 1. The title and abstract of 162 records identified through the initial search were screened following inclusion and exclusion criteria. After deleting unrelated papers, the full-texts of a total of 17 remained articles were checked. In this stage, 10 articles were omitted because of the following reasons: irrelevant articles (*n* = 9) and consideration of both tea and coffee as exposure (*n* = 1). Finally, seven epidemiologic studies possessed eligibility to this study (18–23, 30).

The brief information of each selected study has been described in Table 1. Three cohort studies (18, 19, 30), two case-control studies (20, 22), and two cross-sectional studies (21, 23) were imported to meta-analysis. The studies were published between 2007 and 2021, and these were conducted in the US (18), Denmark (19), Indonesia (22), Oman (20), Spain (21), Malaysia (30), and Ethiopia (23). Women with GDM were aged between 13 and 49 years. A total of 75,607 pregnant women participated, 1,625 of whom were diagnosed with GDM. Three studies used a food frequency questionnaire (FFQ) (18, 21, 30), one 24-h food recall (23), and others used a dietary questionnaire (19, 20, 22). The method of GDM diagnosis was oral glucose tolerance test (OGTT) for four studies (18, 21, 23, 30) and medical records (22) or questionnaire (19, 20) for the others. Four studies controlled the covariates (18, 19, 23, 30), and others did not adjust. Three studies had high-methodological quality (score ≥ 7) (18, 19, 23) (Table 1).

**Table 1:** Characteristics of included studies on the association between coffee intake and gestational diabetes mellitus.

**Author**	**Country**	**Study design**	Age^*^	**Sample size**	**Follow up (years)**	**Cases**	**Outcome assessment**	**Exposure assessment**	**Median/cutoff point**	**RR (95%CI)**	**Quality score**	**Adjustment**
Adeney et al. (18)	US	Cohort	32.1 ± 0.1	576	6	23	3-h OGTT	FFQ/interview	NR	1 0.64 (0.37_1.10)	7	Maternal age, smoking during pregnancy, and regular alcohol use before pregnancy, maternal race, pre-pregnancy BMI, and chronic hypertension
Hinkle et al. (19)	Denmark	Cohort	16–48	71,239	6	912	Discharge Register or self-reported on either of the DNBC interviews	Dietary questionnaire/ interview	0 0.5–3 cups/day 4–7 cups/day ≥8 cups/day	10.97 (0.84-1.13)0.81 (0.64-1.02)0.89 (0.64-1.25)	7	Coffee or tea consumption, age, socio-occupational status, parity, pre-pregnancy body mass index, smoking, and calorie intake
Amiruddin et al. (22)	Indonesia	Case-Control	NR	135	NA	45	Medical record	Dietary history questionnaire/ interview	NR	2.40 (1.10–5.25)	4	No
Chitme et al. (20)	Oman	Case-Control	15–49	591	NA	291	Literature-Based questionnaire	Literature-Based questionnaire/ interview	NR	1 1.12 (0.98–1.29)	2	No
Ramos-Levi et al. (21)	Spain	Cross-Sectional	13–47	2,194	NA	213	OS +OGTT	FFQ/self-report	0–1 cup/day >3 cup/day	1 0.7 (0.4–1.05)	5	No
Larebo et al. (23)	Ethiopia	Cross-Sectional	18–49	420	NA	110	OGTT	24 h food recall/ interview	NR	2.70 (1.04–7.00) 1	9	NR
Yong et al. (30)	Malaysia	Cohort	30.01 ± 4.48	452	NR	31	OGTT	FFQ	NR	1 0.99 (0.98–1.04)	6	Age, parity, total energy, pre-pregnancy BMI, and total gestational weight gain

*RR, Relative Risk; CI, confidence interval; FFQ, food frequency questionnaire; US, United States; NR, not-reported; NA, Not applicable; wk, week; OS, O'Sullivan test; OGTT, Oral glucose tolerance test*.

* *Presented as mean or range*.

### Meta-Analysis

**Figure 2:**
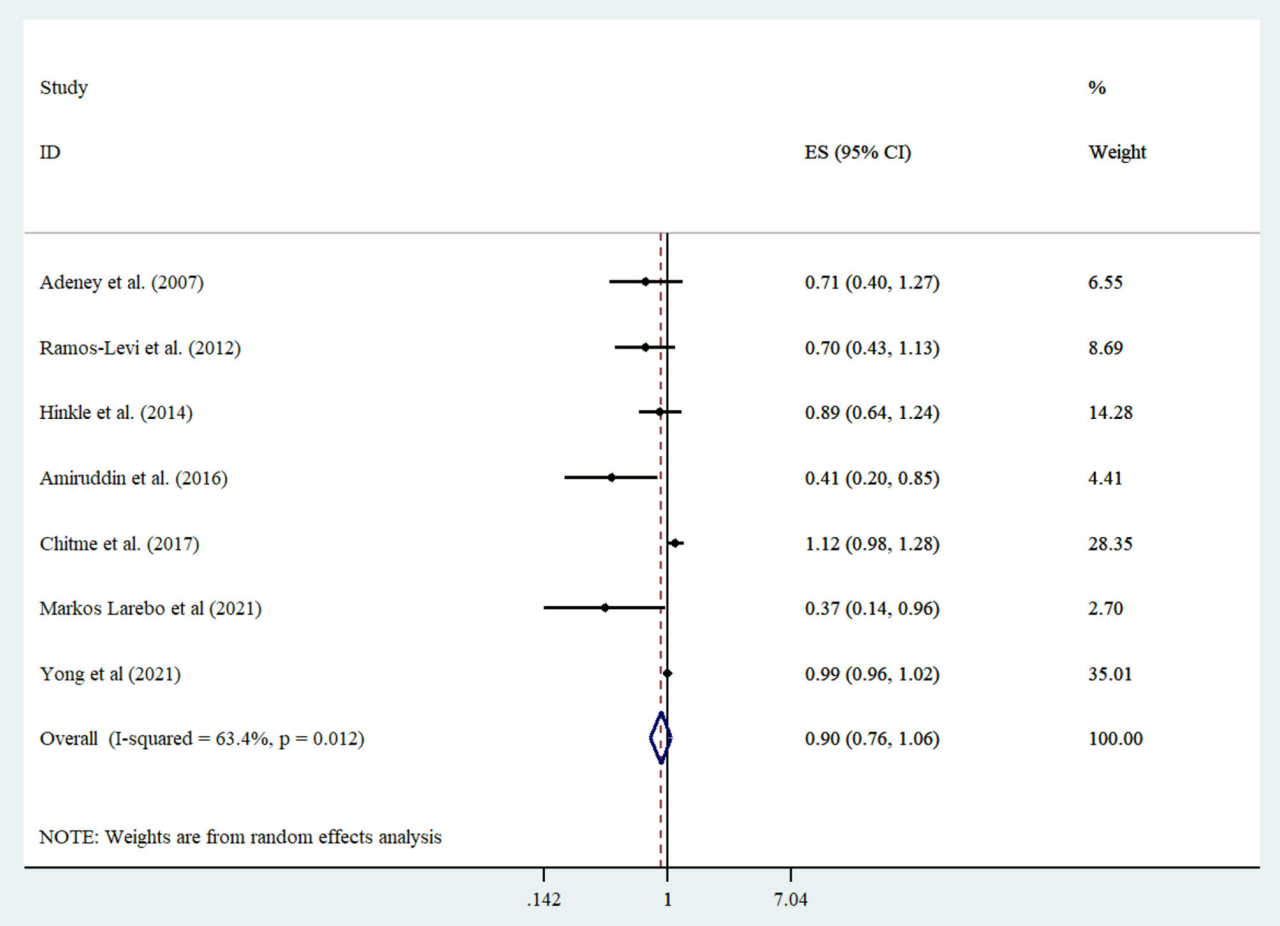
Forest plot derived from random effects meta-analysis of studies investigating the association between high vs. low intake of consumption of coffee and gestational diabetes mellitus. CI, confidence interval; ES, effect size.

Three cohorts, two case-control, and two cross-sectional studies were included in the meta-analysis (18–23, 30). When extreme categories were compared, no significant association was detected between intake of coffee and risk of GDM (summarized risk estimate: 0.89; 95% CI: 0.76, 1.05; *I*^2^ = 63.4%) (Figure 2). Subgroup analysis found study design, study location, sample size, dietary assessment tool, study quality, adjustment, and controlling for energy and BMI as sources of heterogeneity (Table 2). Furthermore, subgroup analysis showed that consumption of coffee had an inverse relationship with GDM in studies conducted in non-Asia countries (Table 2). Sensitivity analysis was performed to assess the effect of each study on overall effect size (Supplementary Figure 1). A study performed by Amiruddin et al. (22) considered both GDM and prediabetes as the outcome. Therefore, we excluded it and carried out the analysis. No significant association was found (summarized risk estimate: 0.95; 95% CI: 0.83, 1.09; *I*^2^ = 53.6%). Furthermore, it seems that studies of Chitme et al. (20) and Yong et al. (30) had an influence on overall effect size. After removing these studies step by step and reanalyzing, we observed that high-coffee intake significantly decreased GDM (summarized risk estimate: 0.75; 95% CI: 0.57, 0.98; *I*^2^ = 62.1%), and marginally decline risk of GDM (summarized risk estimate: 0.73; 95% CI: 0.53, 1.02; *I*^2^ = 71.6%) compared to women with low intake, respectively. We did not identify the evidence of publication bias by Egger test (*P* = 0.116) and inspection of funnel plot (Supplementary Figure 2).

**Table 2:** Summary risk estimates for the association between coffee consumption and risk gestational diabetes mellitus in subgroup analysis^a^.

	**#RR^**b**^**	**Pooled RR (95% CI)^**c**^**	***I^**2**^* (%)^**d**^**	***P*-heterogeneity^**e**^**
**The highest vs. lowest comparison**				
** Coffee intake**				
Overall	7	0.89 (0.76–1.05)	63.4	0.012
**Subgroup analysis**				
**Study location**				
Asia	3	0.99 (0.82–1.19)	76.8	0.013
Non-Asia	4	0.75 (0.58–0.97)	6	0.363
**Study design**				
Cohort	3	0.98 (0.96–1.01)	0	0.439
Case-control	2	0.72 (0.27–1.92)	85.8	0.008
Cross-sectional	2	0.58 (0.33–1.02)	26.4	0.244
**Sample size**				
≤ 1,000	5	0.63 (0.35–1.14)	79.8	0.002
>1,000	2	0.82 (0.62–1.08)	0	0.423
**Number of cases**				
≤ 200	4	0.64 (0.39–1.06)	75.2	0.012
>200	3	0.95 (0.73–1.23)	55.5	0.106
**Dietary assessment tool**				
FFQ	3	0.88 (0.69–1.12)	38.2	0.199
Non-FFQ	4	0.74 (0.48–1.15)	76.3	0.005
**Outcome assessment**				
OGTT	4	0.77 (0.54–1.08)	58.7	0.064
Medical record/questionnaire	3	0.85 (0.57–1.27)	75.5	0.017
**Adjustment**				
Yes	4	0.87 (0.68–1.10)	47.3	0.128
No	3	0.74 (0.43–1.30)	79.9	0.007
**Adjustment for energy intake**				
Yes	3	0.98 (0.96–1.01)	0	0.439
No	3	0.74 (0.43–1.30)	79.9	0.007
NR	1	0.37 (0.14–0.96)	–	–
**Adjustment for BMI**				
Yes	3	0.98 (0.96–1.01)	2.1	0.312
No	3	0.74 (0.43–1.30)	79.9	0.007
NR	1	0.37 (0.14–0.96)	–	–
**Study quality**				
High	3	0.73 (0.49–1.08)	33.9	0.220
Low	4	0.95 (0.79–1.14)	71.8	0.014

a *BMI, body mass index; CI, confidence interval; RR, Relative Risk; FFQ, food frequency questionnaire; OGTT, oral glucose tolerance test*.

b *Number of risk estimates*.

c *Obtained from the random-effects model*.

d *Inconsistency, the percentage of variation across studies due to heterogeneity*.

e *Obtained from the Q-test*.

## Discussion

This meta-analysis of seven observational studies demonstrated that intake of coffee is not linked with GDM. However, a significant inverse association was indicated between intake of coffee and GDM in studies conducted in non-Asia countries. To date, no systematic review and meta-analysis addressing a correlation between intake of coffee and GDM.

Coffee is a common drink all over the world and consists of a combination of antioxidants and micronutrients, which possess favorable effects on cardiovascular disease (31, 32). In this review, we saw no significant reduced risk of GDM among high-coffee consumers compare to those with low intake. Inline with our finding, a cross-sectional study conducted on 785 adult pregnant women in São Paulo illustrated no association between consumption of coffee and tea and GDM (25). In 2018, one meta-analysis investigated the association of poly-phenol-rich foods and the risk of gestational diabetes. This study captured no connection between non-alcoholic beverages (coffee, tea, and juice) and GDM (33). On the other hand, the evidence shows a relationship between GDM and T2DM, and women with GDM are prone to T2D in the future (7). A recent meta-analysis of 30 cohort studies depicts that the risk of T2D lowered by 6% for each cup-per-day increment in consumption of coffee (34). Furthermore, one cup increase of caffeinated and decaffeinated consumption of coffee in a day leads to a 7 and 6% reduced risk of diabetes, respectively (13). Moreover, a clinical trial study found that intake of caffeine was related to impaired insulin sensitivity in women with GDM early in their third trimester but not in controls without GDM (17).

Some potential reasons can be considered for these inconsistencies. First, intake of caffeine during pregnancy has been related to adverse outcomes including low birth weight, elevated risk of delivering, impaired fetal length growth, and an infant with small-for-gestational-age (35–37). According to the guidelines of the American College of Obstetrics and Gynecology (ACOG), daily intake of up to two cups of moderate-strength coffee may be safe for pregnant women (38). It has been demonstrated that pregnant women significantly decline their intakes of caffeine, particularly coffee during pregnancy (39, 40). The low intake of coffee among pregnant women in the most included studies may explain finding any significant association. This study showed an inverse association between intake of coffee and risk of GDM among pregnant women who lived in the western countries. People in the western countries consume higher coffee as compared with Asia countries. In the United States, about 25% of women aged between 20 and 29 years, and 46% of women aged between 30 and 39 years, drink coffee daily (41).

Furthermore, studies reported the amount of coffee used as “cups” without expressing the volume of the cup. Therefore, the lack of standardization in the measurement of coffee must be taken into account. Besides, coffee is commonly drunk along with sugar, creamer, or milk, which attenuates the beneficial impact of coffee on GDM since fructose worsens hepatic insulin resistance (42). Finally, the degree of roasting and the method of preparation of coffee including coffee-grind setting and brew type may be different in varied populations.

Caffeine and its metabolites such as paraxanthine accumulate in the blood during pregnancy because of a decrease in the metabolism of caffeine (14). High concentrations of coffee and its metabolites have been associated with elevated insulin resistance during pregnancy (15). Therefore, it appears that the favorable effect of coffee on GDM may originate from components of non-caffeine, including micronutrients and phenolic compounds, which have antioxidant and prebiotic traits (16). The evidence indicated that coffee constitutes 60%, or nearly 11.1 mmol, of the total intake of daily antioxidant (43). Chlorogenic acid is the main polyphenol in coffee and is assumed to be the main antioxidant of coffee (44). Coffee also contains potassium, magnesium, niacin, and other antioxidants, which may have beneficial effects on glucose metabolism and insulin resistance (45). Furthermore, habitual intake of coffee declines subclinical inflammation and augments adiponectin levels (46, 47) that may improve insulin sensitivity (48).

This study has some limitations that should be considered once we interpret the findings. First, the small number of studies and a moderate amount of heterogeneity among them are the main limitations. We tried to find the sources of heterogeneity through subgroup analysis. Second, four of the seven included studies have case-control or cross-sectional designs. These kinds of studies possess some biases such as recall and selection biases. Third, measurement errors may occur when estimating consumption of coffee, particularly for those with low intake. Fourth, studies applied different methods for the diagnosis of GDM. Fifth, some studies did not control covariates, or they may ignore to adjust some underlying residual confounding. For example, only three studies controlled energy intake, which is an important risk factor for GDM. Moreover, most studies did not have any information on the intake of coffee of women before pregnancy. Finally, a lack of control for these dietary factors could disable us to identify a firm finding.

In conclusion, this systematic review and meta-analysis of seven observational studies have depicted no relationship between extreme intake of coffee and the risk of GDM. Furthermore, prospective cohort studies with a large sample size are obligatory to understand the relationship between coffee and GDM. Given that, the circulating level of caffeine and its metabolites are different in patients with GDM, future studies are required to examine biomarkers of coffee and its metabolites, including serum caffeine and paraxanthine.

## Author Contributions

JN and PW designed the work and extracted the data. TZ and LL analyzed the data. HP wrote the first draft of the manuscript. All authors critically read and approved the final version of the manuscript.

## Conflict of Interest

The authors declare that the research was conducted in the absence of any commercial or financial relationships that could be construed as a potential conflict of interest.

## Publisher's Note

All claims expressed in this article are solely those of the authors and do not necessarily represent those of their affiliated organizations, or those of the publisher, the editors and the reviewers. Any product that may be evaluated in this article, or claim that may be made by its manufacturer, is not guaranteed or endorsed by the publisher.
