# Supplementary material for: Consumption of Coffee and Risk of Gestational Diabetes Mellitus: A Systematic Review and Meta-Analysis of Observational Studies
Source: Front Nutr. 2021 Sep 20;8:739359. doi: 10.3389/fnut.2021.739359 (PMC8488146; doi:10.3389/fnut.2021.739359)
Supplement: Supplementary file 1 [file Data_Sheet_1.docx]

**Supplementary figure 1**

Sensitivity analysis of observational studies assessing the association between coffee consumption and risk of gestational diabetes mellitus. CI, confidence interval

**Supplementary figure 2**

**
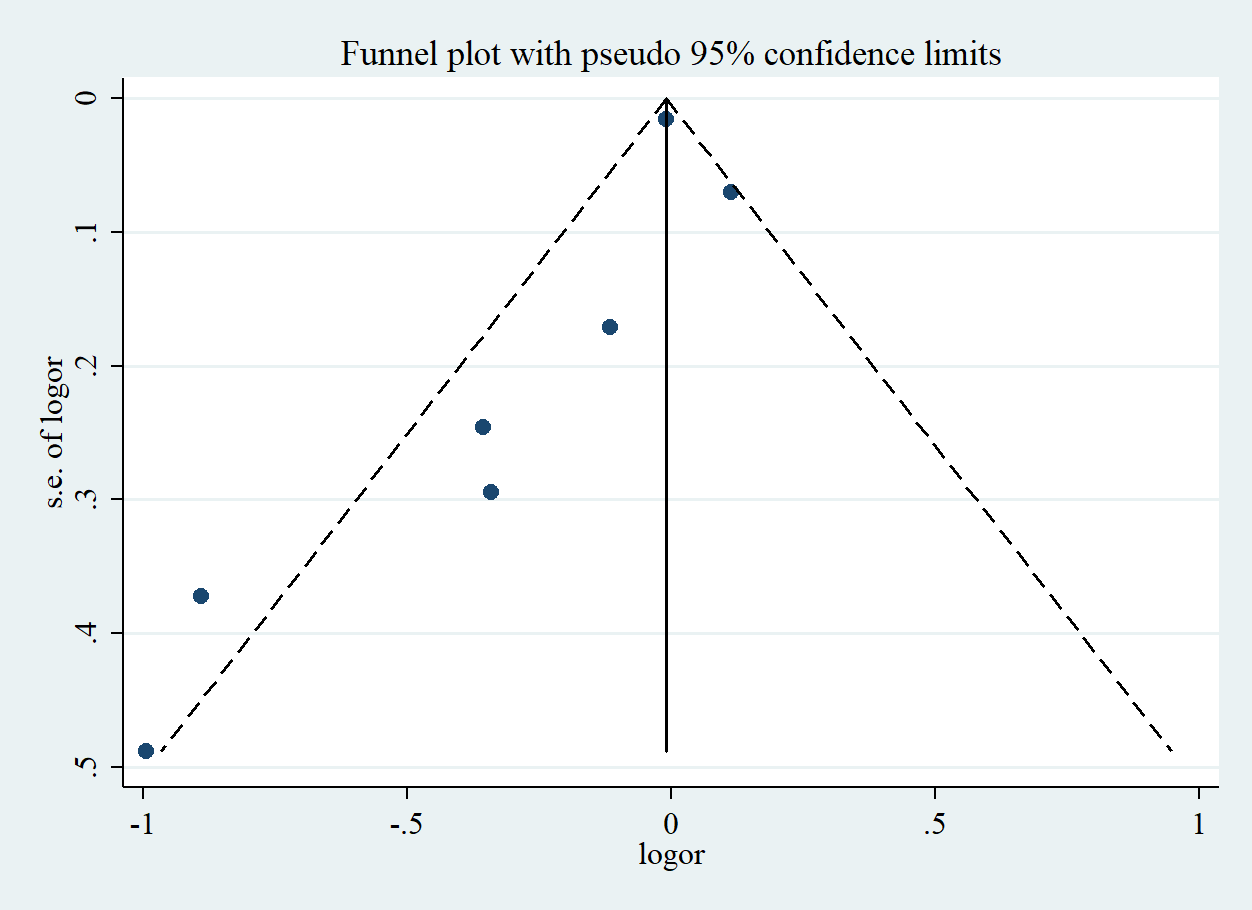
**

Funnel plot of the risk estimates of six studies on the association between coffee consumption and risk of GDM. Each dot represents one study. Egger’s test P<0.001. Log RR: natural logarithm of relative risk. CI, confidence interval; GDM, gestational diabetes mellitus; SE, standard error.
